# The temporal structure of male freestyle wrestling bouts in 65, 86 and 125 kg categories

**DOI:** 10.1371/journal.pone.0282952

**Published:** 2023-03-23

**Authors:** Alfonso Gutiérrez-Santiago, Christopher Vázquez-Estévez, Adrián Paramés-González, Juan Carlos Argibay-González, Xoana Reguera-López-de-la-Osa, Nerea Vila-Fernández, Ana González-Sabajanes, Iván Prieto-Lage

**Affiliations:** 1 Observational Research Group, Faculty of Education and Sport, Universidade de Vigo, Pontevedra, Spain; 2 Education, Physical Activity and Health Research Group (Gies10-DE3), Galicia Sur Health Research Institute (IIS Galicia Sur), SERGAS-UVIGO, Vigo, Spain; Universita degli Studi di Milano, ITALY

## Abstract

In freestyle wrestling, how regulatory breaks and micro pauses affect the efforts during combat has been studied very little. The objective of the study was to determine the temporal structure of fights in male freestyle wrestling in the 65, 86 and 125 kg categories. All wrestlers from the categories (n = 115) who competed in the 2019 senior wrestling world championship (Nur-Sultan, Kazakhstan) participated. Using observational methodology, we analyzed all fights (n = 127). We used different statistical techniques: descriptive, normality tests, Kruskall-Wallis, one-way ANOVA and chi-square. The significance level was ρ<0.05. The results show that most fights finish in the last minute (73.5% in 65 kg, 74.5% in 86 kg and 80.6% in 125 kg) and the total fight time is consumed (67.3% in 65 kg, 70.2% in 86 kg and 77.4% in 125 kg). Differences in 18 variables were found when comparing the three weights were found in the temporal and sequential parameters of the combat. When we compare these parameters to the different fight minutes, we find that there are differences in 17 variables in 65 kg, 20 variables in 86 kg and 10 variables in 125 kg. The results define a temporal structure of male freestyle wrestling bouts in the three categories, and therefore, it will be possible to prepare adequate trainings for these athletes. We conclude that in the three weight categories, they wrestle longer standing than while on the ground. There are clear differences between the three categories. In the 125 kg category, the temporal and sequential parameters are more stable throughout the different minutes of the fight, and in 65 and 86 kg, there is an instability. In the three weights, the regulatory break modulates the duration of the pauses and the actions performed by the wrestlers in different fight minutes.

## Introduction

Olympic wrestling has undergone numerous changes regarding fight duration [1]. In 1896, there was no time limit. In 1913, bouts were limited to two 30-minute periods. In the same year, they decreased the periods to 20 minutes. In 1921, a single 20-minute period was established. There were 15 minutes of combat before 1957. From 1957 to 1961, fights could have lasted up to 12 minutes. From 1962 to 1968, there were two periods of five minutes. From 1969 to 1977 there were three periods of three minutes. From 1978 to 1988, two periods of 3 minutes. From 1989 to 1997, a period of five minutes (plus three minutes of extra time if necessary). From 1998 to 2004, two periods of three minutes (plus three minutes of extra time if necessary). In 2005, two out of the two-minute three periods had to be won. In 2013, the bout was divided into two periods of three minutes, with a regulatory break of 30 seconds, which is the current situation [2].

Knowing the temporal structure of bout helps to better understand its physical demands [3]. Knowing the effort-pause relationship serves us to evaluate sport-specific skills and their metabolic demands [4]. This effort-pause relationship is very different within combat sports. There are sports where the effort is performed without division of the combat into rounds and without regulatory pauses (e.g., judo). Other sports are divided into rounds (e.g., boxing). Others do not have a division into rounds, but do have a regulatory break in the middle of the bout (e.g., wrestling). In addition, in all of them there is an indeterminate number of micro pauses established by the internal logic of the combat itself, which cause an intermittent and changing effort. Herein lies the complexity of studying the temporality of combat.

Therefore, we consider it necessary to quantify the effort made by the wrestlers, to know how the regulatory breaks affect the efforts made by the wrestlers, and to know the number of micro pauses, their duration, their location and how they influence the development of the bout. All this knowledge makes defining the temporal structure of the bout possible, which is crucial for coaches to plan training sessions according to the effort that their athletes will make in competition [5], for training to be as effective as possible, it must be as similar as possible to the competition [1]. That is why there are so many studies in martial arts [6–9] that have tried to clarify the temporal structure, defining the effort made by the athletes and how it is distributed throughout the combat.

Despite the numerous studies in this regard, there are still sports disciplines in which its study has not been addressed in depth, as is the case of wrestling. Olympic wrestling is a sport where the duration of the bout has a maximum of six minutes, with a regulatory break of 30 seconds in the middle of the bout, at the end of the third minute [2]. The bout may end before the regulation time, either because one of the wrestlers gets a fall (touche) or because they obtain a ten-point advantage (technical superiority). This aspect has already been extensively studied in other combat sports such as judo [10]. Existing studies on wrestling have dealt with the temporality of the bout but with very little depth, providing very generic data, and all of them concluding that more research was needed [3, 11–13]. This allows us to indicate that in this discipline, we still do not have studies that determine a thorough temporal structure of the bout, individualized for each minute of the bout and specific for each weight category.

In order to solve this deficiency, we propose a study with the objective of determining the temporal structure of male freestyle wrestling in the 65, 86 and 125 kg categories, establishing the existing differences between these weight categories, as well as the temporal structure in the different minutes of the bout. The results will allow us to propose models of temporal structure in each category, which will support determining the training load.

## Method

### Design

Observational study to determine the temporal and sequential structure of male wrestlers in the 65, 86 and 125 kg categories [14].

The observational design [15] used is nomothetic (several wrestlers/bouts), follow-up (we analyzed the behaviors of the bout during the entire championship), and multidimensional (there is concurrence of behaviors). From this design we derived a series of decisions about the participants, the observation and registration instruments, and the analysis procedure.

### Participants

The participants were all wrestlers in the 65 kg (44 wrestlers), 86 kg (43 wrestlers) and 125 kg (28 wrestlers) that competed in the freestyle wrestling modality at the 2019 senior world championships held in Nur-Sultan (Kazakhstan). All the bouts in those categories were analyzed. The audiovisual material was obtained from the website of the United World Wrestling [16]. It was an observational study in a natural setting, with public videos and not involving experimentation of any kind, the informed consent of the competitors was not required [17]. The study was approved by the Ethics Committee of the Faculty of Education and Sport Science (University of Vigo, Application 02/0320).

Table 1 shows the bouts analyzed in the present study. We analyzed all the bouts of the 65, 86 and 125 kg categories, which were respectively 49, 47 and 31 bouts.

**Table 1:** Fights analyzed in the study regarding the weight category and competition phase.

Round	Weight Category					
	65 kg		86 kg		125 kg	
	Frequency	Percentage	Frequency	Percentage	Frequency	Percentage
**Qualification**	12	24.5	11	23.4	12	38.7
**1/16 Final**	15	30.6	16	34	0	0
**1/8 Final**	8	16.3	8	17	8	25.8
**1/4 Final**	4	8.2	4	8.5	4	12.9
**Semifinal**	2	4.1	2	4.3	2	6.5
**Repechage**	5	10.2	4	8.5	2	6.5
**Bronze**	2	4.1	2	4.3	2	6.5
**Final**	1	2	0	0	1	3.2
Total	49	100	47	100	31	100

### Instruments

The observation instrument developed *ad hoc* for this study is based on the instrument “*Time-Motion Analysis Model in Wrestling*” [4]. Our instrument combines the field format with the category system. This type of combination has been used in multiple studies [18, 19]. It is formed by a set of criteria that allows determining the temporality and sequentiality of freestyle wrestling bout behaviors (see Table 2).

**Table 2:** Observational instrument.

Criteria	Category	Code	Description
Weight Category	65 kg	65	65 kilos category
	86 kg	86	86 kilos category
	125 kg	125	125 kilos category
Moment in the Combat	1st minute	1M	Between 0” and 60”
	2nd minute	2M	Between 61” and 120”
	3rd minute	3M	Between 121” and 180”
	4th minute	4M	Between 181” and 240”
	5th minute	5M	Between 241” and 300”
	6th minute	6M	Between 301” and 360”
Points	No points	NOPT	Techniques or falls that fail to score
	1 point	1PT	Techniques or falls that score with one point
	2 points	2PT	Techniques or falls that score with two points
	4 points	4PT	Techniques or falls that score with four points
Passivities	Passivity	PAS	Start of passivity
	1st Passivity—Score	PAS1PT	1st passivity that scores
	1st Passivity—No Score	PAS1NOPT	1st passivity that does not score
	2nd Passivity—Score	PAS2PT	2nd passivity that scores
	2nd Passivity—No Score	PAS2NOPT	2nd that does not score
	Remaining	Etc.	Until the maximum passivities occur
Standing Fight Sequences	1st standing sequence	STD1	1^st^ sequence of a standing fight
	2nd standing sequence	STD2	2^nd^ sequence of a standing fight
	3rd standing sequence	STD3	3^rd^ sequence of a standing fight
	Remaining	Etc.	Until the maximum sequences occur
Standing actions	Separation	SEPA	Approximate, separation and contact actions
	Grip	GRIP	Gripping actions during the bout
	Hold	HOLD	Holding actions during the bout
	Fall	FALL	Fall that score
	Throw	THROW	Throws
	Push-out	PUSHOUT	Push-out the opponent from the fight zone
	Non-ending attack	ATTSTD	Attempted fall that does not achieve its purpose
Ground Fight Sequence	1st ground sequence	GND1	1st ground fight sequence
	2nd ground sequence	GND2	2^nd^ ground fight sequence
	3rd ground sequence	GND3	3^rd^ ground fight sequence
	Remaining	etc.	Until the maximum sequences occur
Ground actions	Preparation	PREP	Ground preparation sequence
	Attack	ATT	Ground attack sequence that can score or not score
	Transition	TRANS	Ground fight time where no action is being attempted
Pause Sequence	1st pause sequence	PAU1	1st pause sequence
	2nd pause sequence	PAU2	2nd pause sequence
	3rd pause sequence	PAU3	3rd pause sequence
	Remaining	Etc.	Until the maximum sequences occur
Pause Type	Normal	NOR	Pause determined by the rules
	Video	VID	Pause from the referees to review video
	Medical	MED	Paused taken for medical intervention
	Challenge	CHA	Pause taken because of a challenge from the trainer
	Break	BK	30-second regulatory break

The observation instrument conforms to observational design and meets the conditions of exhaustiveness and mutual exclusivity [14]. The construct validity of the observation instrument was carried out through its consistency with the theoretical framework [20] and by consulting two experts in observational methodology and combat sports who had to show their degree of agreement with the instrument, reaching a level of agreement of 96%. After discussion between the two experts and consensus on the discrepant categories, a level of agreement of 100% was reached.

Software LINCE PLUS was used to register the data [21]. This software is a multimedia interactive program that allows the simultaneous viewing and registering of the filmed material in a computer, which also makes the data collection easier.

### Study variables

The variables analyzed in this study have been similar to those of other studies where the bout temporal structure was analyzed. [5, 22]. The study variables were obtained through an observational instrument created for this study ad hoc (Table 1). This instrument is incorporated in the LINCE PLUS software to measure the variables, obtaining the temporality and occurance of the registered variables. The variables analyzed are the following:

- Weight category: 65 kg, 86 kg and 125 kg. These variables are nominal.
- Moment: the moment in which the bouts end (1º, 2º, 3º, 4º, 5º o 6º minute). These variables are nominal.
- Consumption: consumption of the bout time (bouts that consume the total combat time—six minutes- and combats that finish before time is up). These variables are nominal.
- Bout parameters: the sequential and temporal parameters of the freestyle wrestling bouts. All these variables are scalar and are as follows: Total Combat Time, Total fight time (without pauses), Total Standing Time, Total Ground Time, Total Pause Time, Total Passivity Time, Total Standing Separation Time, Total Standing Grip Time, Total Standing Hold Time, Total Standing Fall Time, Total Standing Throw Time, Total Standing Push-out Time;, Total Standing Attack Time, Total Ground Preparation Time, Total Ground Attack Time, Total Ground Transition Time, Total Normal Pause Time TVID, Total Video Pause Time, Total Video Pause Time, Total Medical Pause Time, Total Challenge Pause Time, Total Break Pause Time, Total Standing Sequences, Total Ground Sequences, Total Pause Sequences, Total Normal Pause Sequences, Total Video Pause Sequences, Total Medical Pause Sequences, Total Challenge Pause Sequences, Total Break Pause Sequences, Standing Sequence Time, Ground Sequence Time, Pause Sequence Time, Normal Pause Sequence Time, Video Pause Sequence Time, Medical Pause Sequence Time, Challenge Pause Sequence Time, Break Pause Sequence Time, Total Number of Standing Separation, Total Number of Standing Separation, Total Number of Standing Grip, Total Number of Standing Hold, Total Number of Standing Fall, Point, Total Number of Standing Throw, Total Number of Standing Push-out, Total Number of Standing Attack, Total Number of Ground Preparation, Total Number of Ground Attack, Touche, Total Number of Ground Transition, Total Number of Passivities, Standing Separation Sequence Time, Standing Grip Sequence Time, Standing Hold Sequence Time, Standing Fall Sequence Time, Standing Throw Sequence Time, Standing Push-out Sequence Time, Standing Separation Sequence Time, Standing Attack Sequence Time, Ground Preparation Sequence Time, Ground Attack Sequence Time, Ground Transition Sequence Time, Passivity Sequence Time.

### Procedure

After adequate training in the use of the registration instrument and the observational instrument, rigor in the coding process was guaranteed [23] by controlling the quality of the data to be registered with two expert observers in wrestling by calculating intra- and inter- observer agreement, using Cohen’s Kappa coefficient [24] and calculated using LINCE software [5, 19, 25]. Both concordances were performed with bouts not belonging to the final sample, in a number equivalent to one third of the final sample (n = 42). In the intraobserver concordance, a mean kappa value of 0.90 was obtained for observer 1 and 0.88 for observer 2, and in the interobserver concordance, a mean kappa value of 0.83 was obtained. Subsequently, the data was registered by observer 1 using the LINCE PLUS software.

After registering all the bouts, we obtained an Excel file with the sequentiality and temporality of all the behaviors under study. The versatility of this file allowed us to perform successive transformations for the different analyses [26].

After obtaining the results, we made some models of the temporal structure of the bout. These models were made by consensus by an expert in wrestling and by another expert in combat sports and time-motion with ample experience in the construction of other temporal structure models [5, 26].

### Data analysis

All statistical analyses were performed using IBM- Statistical Package for the Social Sciences, version 20.0 (IBM-SPSS Inc., Chicago, IL, USA). A descriptive analysis, stratified by weight categories, was carried out for each of the variables under study: through frequencies and percentages for qualitative variables, and through measures of central tendency (mean, standard deviation and 95% confidence intervals) for quantitative variables. The normality of the sample was tested using the Kolmogorov-Smirnov test (with the Lilliefors correction) in variables in more than 50 cases and with Shapiro-Wilk in the variables of 50 cases or less. The average values of the sequential and temporal parameters of the freestyle wrestling bouts were compared among three weight categories and between the different minutes that ended the bout through an ANOVA of one way (applying a post hoc Tukey-b test in case there were any significant statistical differences) when the sample was normal or through the Kruskall-Wallis test when the sample was not normal. The relationship between the qualitative categories and the comparison of these qualitative categories among the three weight categories was studied using the chi-square test. In all statistical tests, p < 0.05 was considered as the level of significance.

## Results

### General statistical analysis of the bout

Regarding the moment in which the bouts end, in the 65 kg category, 4.1% (n = 2) of the bouts end in the first and second minute, 6.1% (n = 3) in the third minute, 4.1% (n = 2) in the fourth minute, 8.2% (n = 4) in the fifth minute and 73.5% (n = 36) in the last minute, with significant differences between the different minutes (χ2 = 114.224, p = 0.000). In the 86 kg, 2.1% (n = 1) of the bouts end in the first minute, 4.3% (n = 2) in the second minute, 10.6% (n = 5) in the third minute, 4.3% (n = 2) in the fourth and fifth minute and 74.5% (n = 35) in the last minute, with significant differences between the different minutes (χ2 = 114.234, p = 0.000). In the 125 kg category, 6.5% (n = 2) of the bouts end in the second minute, 9.7% (n = 3) in the fourth minute, 3.2% (n = 1) in the fifth minute and 80.6% (n = 25) in the last minute, with significant differences between the different minutes (χ2 = 51.452, p = 0.000). When we compare the three categories, we find that there are no significant differences (χ2 = 7.465, p = 0.681) since most of the bouts end in the last minute in the three weights.

We checked whether the bouts generally consumed the total time of the bout (six minutes) or ended before time. In the 65 kg category, 67.3% (n = 33) consumed the total time of the bout and 32.7% (n = 16) did not consume the total time of the bout, with significant differences between both variables (χ2 = 5.898, p = 0.015). In the 86 kg category, 70.2% (n = 33) do consume the total time of the bout and 29.8% (n = 14) do not consume it with significant differences between both variables (χ2 = 7.681, p = 0.006). In 125 kg, 77.4% (n = 24) do consume the total time of the bout and 22.6% (n = 7) do not consume it, with significant differences between both variables (χ2 = 9.323, p = 0.002). When comparing this aspect among the three weight categories, we observed that there are no significant differences (χ2 = 0.948, p = 0.622) since the distribution of the variables is similar in the three weights.

Table 3 shows the sequential and temporal parameters of the freestyle wrestling bouts in the three weight categories. The comparison of the three categories indicates that significant differences were found in up to 18 variables. We will further discuss this aspect in the discussion.

**Table 3:** Sequential and temporary parameters in the 65, 86 and 125kg categories.

Categories	Nor	65kg					86 kg					125 kg					ANOVA or Kruskal-Wallis		
					95% CI					95% CI					95% CI				
		N	Mean	SD	Low	Up	N	Mean	SD	Low	Up	N	Mean	SD	Low	Up	F o H	gl	p
**TCT**	NO	49	456.63	158.36	411.15	502.12	47	421.83	146.32	378.87	464.79	31	458.77	106.07	419.87	497.68	1.654	2	0.437
**TFT**	NO	49	312.98	93.93	286.00	339.96	47	309.49	93.71	281.98	337.00	31	331.65	73.36	304.74	358.56	2.429	2	0.297
**TSTDT**	NO	49	253.69	91.26	227.48	279.91	47	270.34	93.39	242.92	297.76	31	297.42	81.06	267.69	327.15	7.687	2	0.021
**TGNDT**	NO	49	58.78	39.76	47.36	70.19	45	41.62	35.92	30.83	52.42	29	36.62	25.71	26.84	46.40	10.271	2	0.006
**TPT**	NO	47	148.91	85.45	123.83	174.00	46	114.28	71.20	93.14	135.43	30	132.87	62.66	109.47	156.27	4.474	2	0.107
**TPAT**	NO	24	43.71	21.75	34.52	52.89	24	43.75	24.98	33.20	54.30	18	53.28	27.52	39.59	66.97	2.330	2	0.312
**TSEPAT**	NO	49	78.47	49.12	64.36	92.58	47	52.00	29.31	43.39	60.61	31	55.90	26.01	46.36	65.44	9.293	2	0.010
**TGRIPT**	SÍ	49	128.82	65.27	110.07	147.56	47	175.21	72.00	154.07	196.35	31	206.94	75.45	179.26	234.61	12.483	2	0.000
**THOLDT**	NO	45	36.00	28.49	27.44	44.56	45	31.80	25.60	24.11	39.49	29	25.93	21.06	17.92	33.94	3.037	2	0.219
**TFALLT**	NO	49	8.02	5.76	6.37	9.68	44	9.25	7.50	6.97	11.53	30	5.83	5.13	3.92	7.75	6.187	2	0.045
**TTHROWT**	NO	10	1.43	.79	0.86	2.00	4	2.50	1.73	-0.26	5.26	0					1.644	1	0.200
**TPUSHOUTT**	NO	26	3.31	6.06	0.86	5.75	26	1.88	1.28	1.37	2.40	11	2.64	2.06	1.25	4.02	0.836	2	0.658
**TATTSTDT**	NO	36	4.92	4.46	3.41	6.43	31	4.52	3.36	3.29	5.75	20	5.85	5.92	3.08	8.62	0.029	2	0.985
**TPREPT**	NO	43	27.21	20.87	20.79	33.63	39	21.64	13.50	17.27	26.02	25	17.84	13.62	12.22	23.46	4.859	2	0.088
**TATTGNDT**	NO	49	33.45	24.34	26.46	40.44	43	22.72	24.51	15.18	30.26	27	19.15	13.64	13.75	24.54	11.581	2	0.003
**TTRANST**	NO	11	7.16	6.53	2.78	11.55	8	10.38	9.96	2.05	18.70	11	9.09	4.99	5.74	12.44	1.044	2	0.593
**TNORPT**	NO	47	57.62	34.57	47.47	67.77	45	51.51	23.94	44.32	58.70	30	48.70	21.32	40.74	56.66	0.521	2	0.771
**TVIDPT**	NO	5	40.20	4.55	34.55	45.85	4	58.00	19.90	26.34	89.66	1	50.00				3.280	2	0.194
**TMEDPT**	NO	8	116.50	61.85	64.80	168.20	2	64.00	18.38	-101.18	229.18	3	48.67	3.21	40.68	56.65	5.933	2	0.051
**TCHAPT**	NO	8	131.25	109.01	40.12	222.38	6	113.50	18.94	93.62	133.38	7	122.14	54.00	72.20	172.08	0.336	2	0.845
**TBKPT**	NO	42	49.62	8.26	47.04	52.19	40	47.73	6.34	45.70	49.75	29	50.93	8.86	47.56	54.30	2.253	2	0.324
**TSTDS**	NO	49	10.10	3.68	9.04	11.16	47	8.83	3.16	7.90	9.76	31	8.87	3.05	7.75	9.99	5.596	2	0.061
**TGNDS**	NO	49	4.86	2.38	4.17	5.54	45	2.91	2.02	2.30	3.52	30	2.83	1.82	2.15	3.51	21.625	2	0.000
**TPS**	NO	47	8.55	2.98	7.68	9.43	46	7.74	3.21	6.79	8.69	30	7.90	2.55	6.95	8.85	1.779	2	0.411
**TNORPS**	NO	47	7.09	2.79	6.27	7.90	46	6.57	2.76	5.74	7.39	30	6.53	2.39	5.64	7.43	0.894	2	0.639
**TVIDPS**	NO	5	1.00	0.00			4	1.00	0.00			1	1.00				0.000	2	1.000
**TMEDPS**	NO	8	1.50	.53	1.05	1.95	2	1.50	0.71	-4.85	7.85	3	1.00	0.00			2.250	2	0.325
**TCHAPS**	NO	8	1.25	.71	0.66	1.84	6	1.17	0.41	0.74	1.60	7	1.14	0.38	0.79	1.49	0.016	2	0.992
**TBKPS**	NO	42	1.00	0.00			39	1.00	0.00			29	1.00	0.00			0.000	2	1.000
**STDST**	NO	49	26.57	9.95	23.71	29.43	47	32.27	10.35	29.23	35.31	31	36.06	12.46	31.49	40.63	15.981	2	0.000
**GNDST**	NO	49	12.65	6.06	10.91	14.39	45	14.32	8.23	11.85	16.80	29	13.05	5.53	10.95	15.16	0.916	2	0.632
**PST**	NO	47	17.93	12.11	14.37	21.49	46	14.43	6.69	12.45	16.42	30	18.22	11.66	13.87	22.57	5.638	2	0.060
**NORPST**	NO	47	7.73	2.14	7.10	8.36	45	7.91	1.93	7.34	8.49	30	7.42	1.79	6.75	8.09	1.537	2	0.464
**VIDPST**	NO	5	40.20	4.55	34.55	45.85	4	58.00	19.90	26.34	89.66	1	50.00				3.280	2	0.194
**MEDPST**	NO	8	81.88	47.34	42.30	121.45	2	44.75	8.84	-34.66	124.16	3	48.67	3.21	40.68	56.65	5.182	2	0.075
**CHAPST**	SÍ	8	99.42	41.77	64.49	134.34	6	104.17	30.25	72.42	135.91	7	109.07	47.49	65.15	153.00	0.103	2	0.902
**BKPST**	NO	42	49.62	8.26	47.04	52.19	39	48.08	6.01	46.13	50.03	29	50.93	8.86	47.56	54.30	1.737	2	0.420
**TNSEPA**	SÍ	49	27.41	13.49	23.53	31.28	47	26.74	11.10	23.49	30.00	31	27.19	10.57	23.32	31.07	0.038	2	0.963
**TNGRIP**	SÍ	49	26.80	13.15	23.02	30.57	47	26.70	11.51	23.32	30.08	31	27.39	9.94	23.74	31.03	0.035	2	0.966
**TNHOLD**	NO	45	6.47	3.93	5.28	7.65	45	5.36	3.09	4.43	6.28	29	4.59	2.73	3.55	5.63	4.441	2	0.109
**TNFALL**	NO	49	4.80	2.52	4.07	5.52	44	3.48	1.92	2.89	4.06	30	2.87	1.83	2.18	3.55	14.026	2	0.001
**TNFALL No Pt**	NO	47	4.38	2.27	3.72	5.05	37	2.49	1.84	1.87	3.10	22	2.05	1.17	1.52	2.57	26.993	2	0.000
**TNFALL 2 Pt**	NO	15	1.40	.83	0.94	1.86	31	1.61	0.72	1.35	1.88	18	1.89	1.02	1.38	2.40	3.080	2	0.214
**TNFALL 4 Pt**	NO	8	1.00	0.00			10	1.10	0.32	0.87	1.33	6	1.17	0.41	0.74	1.60	1.255	2	0.534
**TNTHRO**	NO	10	1.30	.48	0.95	1.65	4	1.25	0.50	0.45	2.05	0					0.033	1	0.857
**TNTHRO No Pt**	NO	4	1.00	0.00			2	1.00	0.00			0					0.000	1	1.000
**TNTHRO. 4 Pt**	NO	8	1.50	1.41	0.32	2.68	3	1.00	0.00			0					0.375	1	0.540
**TNPUSHOUT**	NO	26	1.73	1.22	1.24	2.22	29	1.45	0.74	1.17	1.73	10	2.50	1.72	1.27	3.73	3.620	2	0.164
**TNATTSTD**	NO	35	1.66	1.03	1.30	2.01	29	1.76	0.87	1.43	2.09	20	1.50	0.83	1.11	1.89	1.794	2	0.408
**TNPREP**	NO	43	4.07	2.90	3.18	4.96	38	3.37	1.87	2.76	3.98	26	3.08	2.43	2.09	4.06	3.617	2	0.164
**TNAGND**	NO	49	6.29	3.49	5.28	7.29	43	3.67	2.71	2.84	4.51	27	3.44	2.17	2.59	4.30	24.052	2	0.000
**TNAGND No Pt**	NO	42	3.00	1.71	2.47	3.53	31	1.84	1.21	1.39	2.28	20	2.15	1.27	1.56	2.74	10.446	2	0.005
**TNAGND 1Pt**	NO	7	1.43	.79	0.70	2.16	4	1.00	0.00			4	1.75	0.50	0.95	2.55	4.029	2	0.133
**TNAGND 2Pt**	NO	45	3.78	2.49	3.03	4.53	33	2.85	1.92	2.17	3.53	20	2.10	1.33	1.48	2.72	10.062	2	0.007
**TNAGND Toc**	NO	4	1.00	0.00			2	1.00	0.00			3	1.00	0.00			0.000	2	1.000
**TNTRANS**	NO	11	1.91	1.30	1.04	2.78	7	1.29	0.76	0.59	1.98	11	1.36	0.67	0.91	1.82	2.053	2	0.358
**TNPA**	NO	24	1.29	.46	1.10	1.49	24	1.96	1.12	1.48	2.43	18	2.00	1.19	1.41	2.59	5.810	2	0.055
**SEPAST**	NO	49	2.95	2.00	2.38	3.53	47	1.91	0.65	1.72	2.10	31	2.12	0.74	1.85	2.39	22.950	2	0.000
**GRIPST**	NO	49	5.17	2.30	4.51	5.83	47	6.99	2.88	6.15	7.84	31	7.84	2.94	6.76	8.91	21.432	2	0.000
**HOLDST**	NO	45	5.65	2.73	4.83	6.47	45	5.73	2.62	4.94	6.51	29	5.18	2.68	4.16	6.20	1.134	2	0.567
**FALLST**	NO	49	1.74	.98	1.46	2.02	44	2.63	1.62	2.14	3.12	30	1.96	0.86	1.64	2.28	11.313	2	0.003
**THROWST**	SÍ	10	1.13	.56	0.73	1.53	4	1.88	0.63	0.87	2.88	0					4.747	1	0.050
**PUSHOUTST**	NO	26	1.91	3.01	0.70	3.12	26	1.30	0.54	1.08	1.51	10	1.44	1.63	0.27	2.61	0.910	2	0.634
**ATTSTDST**	NO	35	3.13	3.21	2.03	4.23	29	2.60	1.15	2.16	3.04	20	4.01	3.85	2.21	5.82	0.616	2	0.735
**PREPST**	NO	43	7.02	2.86	6.14	7.90	38	7.36	4.44	5.90	8.82	25	6.47	3.76	4.91	8.02	0.796	2	0.672
**ATTGNDST**	NO	49	5.56	3.19	4.65	6.48	43	6.31	6.02	4.45	8.16	27	6.38	4.95	4.42	8.34	0.624	2	0.732
**TRANSST**	SÍ	11	3.48	2.00	2.14	4.83	7	5.57	2.76	3.02	8.12	11	6.79	3.07	4.73	8.85	4.403	2	0.023
**PAST**	NO	24	33.92	12.58	28.61	39.23	24	23.94	11.41	19.12	28.75	18	29.49	11.39	23.83	35.15	9.465	2	0.009

Abbreviations: CI = Confidence interval; Nor = Normal; TCT = Total Combat Time; TFT = Total fight time (without pauses); TSTDT = Total Standing Time; TGNDT = Total Ground Time; TPT = Total Pause Time; TPAT = Total Passivity Time; TSEPAT = Total Standing Separation Time; TGRIPT = Total Standing Grip Time; THOLDT = Total Standing Hold Time; TGRIPT = Total Standing Fall Time; TTHROWT = Total Standing Throw Time; TPUSHOUTT = Total Standing Push-out Time; TATTSTDT = Total Standing Attack Time; TPREPT = Total Ground Preparation Time; TATTGNDT = Total Ground Attack Time; TTRANST = Total Ground Transition Time; TNORPT = Total Normal Pause Time; TVIDEOPT = Total Video Pause Time; TVIDPT = Total Video Pause Time; TMEDPT = Total Medical Pause Time; TCHAPT = Total Challenge Pause Time; TBKPT = Total Break Pause Time; TSTDS = Total Standing Sequences; TGNDS = Total Ground Sequences; TPS = Total Pause Sequences; TNORPS = Total Normal Pause Sequences; TVIDPS = Total Video Pause Sequences; TMEDPS = Total Medical Pause Sequences; TCHAPS = Total Challenge Pause Sequences; TBKPS = Total Break Pause Sequences; STDST = Standing Sequence Time; GNDST = Ground Sequence Time; PST = Pause Sequence Time; NORPST = Normal Pause Sequence Time; VIDPST = Video Pause Sequence Time; MEDPST = Medical Pause Sequence Time; CHAPST = Challenge Pause Sequence Time; BKPST = Break Pause Sequence Time; TNSEPA = Total Number of Standing Separation; TNSEPA = Total Number of Standing Separation; TNGRIP = Total Number of Standing Grip; TNHOLD = Total Number of Standing Hold; TNFALL = Total Number of Standing Fall; Pt = Point; TNTHRO = Total Number of Standing Throw; TNPUSHOUT = Total Number of Standing Push-out; TNATTSTD = Total Number of Standing Attack; TNPREP = Total Number of Ground Preparation; TNAGND = Total Number of Ground Attack; Toc = Touche; TNTRANS = Total Number of Ground Transition; TNPA = Total Number of Passivities; SEPAST = Standing Separation Sequence Time; GRIPST = Standing Grip Sequence Time; HOLDST = Standing Hold Sequence Time; FALLST = Standing Fall Sequence Time; THROWST = Standing Throw Sequence Time; PUSHOUTST = Standing Push-out Sequence Time; SEPAST = Standing Separation Sequence Time; ATTSTDST = Standing Attack Sequence Time; PREPST = Ground Preparation Sequence Time; ATTGNDST = Ground Attack Sequence Time; TRANSST = Ground Transition Sequence Time; PAST = Passivity Sequence Time.

### Statistical analysis in each minute of the bout

The previous results indicate that the number of bouts that end in each of the minutes of the bout is not equal, with significant differences between the different minutes in the three weight categories. Thus, in all three weights, most of the bouts end in the last minute of the bout. For these reasons, it is necessary to analyze the temporal and sequential parameters in the different minutes of the bout (1st minute, 2nd minute, 3rd minute, 4th minute, 5th minute and 6th minute).

Tables 4–6 show the sequential and temporal parameters of the freestyle wrestling bouts in the different minutes, in the 65, 86 and 125 kg categories, respectively. Thus, when we compare the different minutes of the fight, we observe that in 65 kg, there are significant differences in 17 temporal and sequential parameters; in 86 kg in 20 variables and in 125 kg in 10 variables. We will go deeper into these differences in the discussion.

**Table 4:** Sequential and temporal parameters in the different bout minutes in the 65 kg category.

Categories	Nor	Moment in the bout												ANOVA or Kruskal-Wallis		
		1st minute		2nd minute		3rd minute		4th minute		5th minute		6th minute				
		Mean	SD	Mean	SD	Mean	SD	Mean	SD	Mean	SD	Mean	SD	F o H	gl	p
**TCT**	NO	67.14	13.05	75.96	21.05	74.22	22.07	74.17	19.14	80.80	29.20	103.81	78.91	26.439	5	0.000
**TFT**	NO	59.39	4.92	60.13	4.18	58.82	11.03	60.60	7.39	59.53	5.05	57.00	12.03	20.213	5	0.001
**TSTDT**	NO	51.14	11.40	49.19	13.80	49.52	14.68	49.74	13.59	46.03	14.04	42.14	15.64	11.690	5	0.039
**TGNDT**	NO	13.47	8.62	18.25	11.13	14.63	10.19	17.54	12.12	17.42	12.93	17.83	12.67	3.506	5	0.622
**TPT**	NO	10.00	11.95	16.17	21.44	19.80	23.48	16.76	16.40	25.03	29.22	54.35	80.58	33.339	5	0.000
**TPAT**	SÍ			18.81	10.99	20.72	14.31	14.00	6.61	22.67	16.15			.698	3	.557
**TSEPAT**	NO	18.57	12.01	13.98	8.18	15.93	11.58	14.86	11.29	13.23	9.47	12.74	7.06	7.661	5	0.176
**TGRIPT**	NO	25.71	12.87	28.09	14.16	27.29	13.07	24.61	9.99	22.20	11.96	21.09	11.62	9.061	5	0.107
**THOLDT**	NO	10.36	8.44	9.00	6.57	9.71	7.27	11.38	8.59	8.27	7.04	9.23	6.55	3.070	5	0.689
**TFALLT**	NO	2.29	1.38	2.55	2.48	3.36	3.74	2.83	3.24	2.71	1.67	2.96	2.58	3.462	5	0.629
**TTHROWT**	NO	1.33	.58	1.50	.71	1.25	.50	1.00		1.15	1.20			0.690	4	0.953
**TPUSHOUTT**	NO	1.25	.50	.90	.19	7.33	12.23	1.38	.74	1.23	.64	1.89	.93	11.311	5	0.046
**TATTSTDT**	NO	2.56	1.81	1.86	.90	2.00	1.10	6.78	7.74	3.47	2.13	2.40	1.07	7.162	5	0.209
**TPREPT**	NO	8.10	5.18	11.50	7.39	10.48	5.63	13.64	7.26	10.64	5.70	11.32	7.50	6.187	5	0.288
**TATTGNDT**	NO	8.48	6.14	11.08	7.96	8.27	6.66	10.54	8.67	10.73	11.07	10.72	8.08	2.860	5	0.722
**TTRANST**	NO	2.00		10.00		2.50	1.80	3.50	.71	6.00	5.66	3.26	3.50	4.568	5	0.471
**TNORPT**	NO	8.97	7.22	11.45	6.07	12.12	10.15	11.55	7.08	14.39	9.62	21.27	17.07	21.874	5	0.001
**TVIDPT**	SÍ					40.50	6.36	42.00	4.24	36.00				.415	2	.707
**TMEDPT**	NO	39.00		79.67	47.06	79.00	25.46	40.00		68.00	18.57	184.00		6.615	5	0.251
**TCHAPT**	NO					54.00		66.00		96.00		172.80	125.80	3.467	3	0.325
**TBKPT**	NO					49.62	8.26	5.00						2.874	1	0.090
**TSTDS**	NO	2.39	1.11	1.93	.88	1.47	.74	2.43	.99	1.89	.96	2.09	1.15	27.486	5	0.000
**TGNDS**	NO	1.33	.55	1.54	.78	1.36	.62	1.27	.53	1.59	.91	1.79	.92	8.172	5	0.147
**TPS**	NO	1.53	.83	1.76	.77	2.09	.89	1.57	.70	1.70	.92	1.91	1.17	13.543	5	0.019
**TNORPS**	NO	1.50	.73	1.77	.77	1.34	.70	1.50	.66	1.67	.92	1.71	1.04	8.613	5	0.126
**TVIDPS**	NO					1.00	0.00	1.00	0.00	1.00				0.000	2	1.000
**TMEDPS**	NO	1.00		1.00	0.00	1.00	0.00	1.00		1.00	0.00	1.00		0.000	5	1.000
**TCHAPS**	NO					1.00		1.00		1.00		1.40	.89	0.600	3	0.896
**TBKPS**	NO					1.00	0.00									
**STDST**	NO	25.60	14.17	30.70	14.99	41.90	19.24	24.22	13.81	32.78	18.63	29.10	19.39	23.967	5	0.000
**GNDST**	NO	10.73	7.06	12.47	9.54	11.64	9.40	15.39	12.03	13.01	11.69	11.18	9.61	3.130	5	0.680
**PST**	NO	5.76	2.62	10.12	13.48	9.28	16.46	10.20	8.11	14.91	17.18	27.16	37.99	55.268	5	0.000
**NORPST**	NO	5.60	2.23	6.73	2.44	8.71	4.21	7.47	3.78	8.52	3.04	11.55	6.23	40.318	5	0.000
**VIDPST**	SI					40.50	6.36	42.00	4.24	36.00				.415	2	.707
**MEDPST**	NO	39.00		79.67	47.06	79.00	25.46	40.00		68.00	18.57	184.00		6.615	5	0.251
**CHAPST**	SÍ					54.00		66.00		96.00		121.87	46.47	.864	3	.529
**BKPST**	NO					49.62	8.26									
**TNSEPA**	NO	6.37	2.89	5.23	2.72	4.81	2.70	5.86	2.66	4.38	2.25	4.54	2.11	16.317	5	0.006
**TNGRIP**	NO	6.13	2.75	5.23	2.54	4.90	2.25	5.69	2.78	4.43	2.37	4.44	2.11	13.013	5	0.023
**TNHOLD**	NO	1.96	1.07	1.62	1.05	1.35	.56	1.97	1.27	1.63	1.10	1.88	.82	12.047	5	0.034
**TNFALL**	NO	1.39	.63	1.42	.58	1.29	.46	1.27	.45	1.69	.93	1.83	.93	9.561	5	0.089
**TNFALL No Pt**	NO	1.36	.57	1.35	.59	1.29	.46	1.17	.39	1.62	.90	1.80	.96	9.700	5	0.084
**TNFALL 2 Pt**	NO	1.50	.71	1.00	0.00	1.00		1.00	0.00	1.00	0.00	1.00	0.00	8.500	5	0.131
**TNFALL 4 Pt**	NO	1.00	0.00			1.00		1.00	0.00			1.00	0.00	0.000	3	1.000
**TNTHRO**	NO	1.00	0.00	1.50	.71	1.00	0.00	1.00		1.00	0.00			5.000	4	0.287
**TNTHRO No Pt**	NO	1.00		1.00	0.00					1.00				0.000	2	1.000
**TNTHRO. 4 Pt**	NO	1.00	0.00	1.00		1.00	0.00	1.00		4.00				8.000	4	0.092
**TNPUSHOUT**	NO	1.00	0.00	1.00	0.00	1.29	.49	1.22	.44	1.00	0.00	1.00	0.00	7.193	5	0.207
**TNATTSTD**	NO	1.00	0.00	1.00	0.00	1.00	0.00	1.33	.50	1.21	.58	1.10	.32	7.186	5	0.207
**TNPREP**	NO	1.38	.74	2.06	1.26	1.62	.74	1.79	1.31	1.53	.62	1.53	.84	5.439	5	0.365
**TNAGND**	NO	1.59	.98	2.40	1.44	1.67	.84	1.60	.96	2.13	1.11	1.93	1.05	10.870	5	0.054
**TNAGND No Pt**	NO	1.27	.59	1.41	1.00	1.38	.50	1.21	.43	1.59	.71	1.38	.50	4.381	5	0.496
**TNAGND 1Pt**	NO	1.00		2.00		1.00		1.00		1.00		2.00	1.41	3.333	5	0.649
**TNAGND 2Pt**	NO	1.56	1.09	2.33	1.35	1.30	.66	1.40	.74	1.59	.91	1.33	.58	13.076	5	0.023
**TNAGND Toc**	NO	1.00				1.00		1.00		1.00				0.000	3	1.000
**TNTRANS**	NO	1.00		1.00		1.00	0.00	1.00	0.00	1.40	.89	1.20	.45	1.500	5	0.913
**TNPA**	NO			1.00	0.00	1.00	0.00	1.00	0.00	1.00	0.00			0.000	3	1.000
**SEPAST**	NO	3.47	4.29	3.15	2.79	4.61	9.22	2.70	2.46	3.19	2.71	3.29	2.41	4.053	5	0.542
**GRIPST**	NO	5.02	4.28	6.05	4.01	6.60	4.81	4.88	3.11	6.00	4.61	5.14	3.11	8.425	5	0.134
**HOLDST**	NO	5.38	3.21	5.68	3.72	6.76	5.02	6.23	4.82	5.49	4.71	5.17	4.06	2.695	5	0.747
**FALLST**	NO	1.24	1.06	1.76	2.16	2.19	1.78	1.96	1.75	1.81	1.31	1.43	1.20	5.673	5	0.339
**THROWST**	NO	1.33	.58	1.00	0.00	1.25	.50	1.00		1.15	1.20			0.683	4	0.953
**PUSHOUTST**	NO	1.25	.50	.90	.19	4.00	5.60	1.00	.25	.98	.57	1.89	.93	12.283	5	0.031
**ATTSTDST**	NO	2.56	1.81	1.86	.90	2.00	1.10	5.22	5.82	3.21	2.15	2.20	.92	3.718	5	0.591
**PREPST**	NO	6.39	5.02	6.42	3.99	7.07	3.42	9.16	4.62	7.42	3.90	7.64	4.54	4.094	5	0.536
**ATTGNDST**	NO	5.98	5.01	5.60	5.04	5.49	4.95	7.95	8.45	5.59	7.44	5.84	4.71	4.668	5	0.458
**TRANSST**	SI	2.00		10.00		2.50	1.80	3.50	.71	4.11	1.88	2.40	1.78	3.580	5	.033
**PAST**	SI			18.81	10.99	20.72	14.31	14.00	6.61	22.67	16.15			.698	3	.557

Note: Abbreviations in Table 3.

**Table 5:** Sequential and temporal parameters in the different minutes of the bout in the 86 kg category.

Categories	Nor	Moment in the bout												ANOVA or Kruskal-Wallis		
		1st minute		2nd minute		3rd minute		4th minute		5th minute		6th minute				
		Mean	SD	Mean	SD	Mean	SD	Mean	SD	Mean	SD	Mean	SD	F o H	gl	p
**TCT**	NO	72.19	19.10	74.63	16.94	65.45	17.45	66.59	10.84	67.19	14.98	91.83	54.64	16.471	5	0.006
**TFT**	NO	61.09	1.18	59.30	5.78	57.73	13.27	59.31	7.94	57.73	10.45	57.89	10.86	26.665	5	0.000
**TSTDT**	NO	54.43	12.39	53.87	11.68	51.73	13.95	53.79	11.23	51.49	13.83	42.63	17.99	19.564	5	0.002
**TGNDT**	NO	16.47	13.84	13.89	8.33	14.67	9.86	13.44	7.69	19.65	13.91	22.25	14.85	5.769	5	0.329
**TPT**	NO	11.86	19.46	17.63	14.59	11.76	9.18	9.79	4.93	12.50	6.72	39.60	54.32	30.138	5	0.000
**TPAT**	NO			22.95	11.55	17.88	14.83	21.20	14.25	20.50	16.00	21.60	8.47	1.854	4	0.763
**TSEPAT**	NO	12.47	6.77	9.60	5.42	11.50	6.08	10.10	6.68	9.68	9.41	8.64	6.11	14.552	5	0.012
**TGRIPT**	NO	37.51	12.52	34.83	13.69	33.12	11.29	36.00	11.95	33.97	14.38	23.77	12.34	25.117	5	0.000
**THOLDT**	NO	6.00	5.67	10.33	7.70	9.41	6.93	9.27	6.56	7.75	6.94	12.61	10.86	12.225	5	0.032
**TFALLT**	NO	2.35	1.42	3.23	1.97	3.25	2.83	3.47	3.32	5.44	4.94	2.78	3.74	10.911	5	0.053
**TTHROWT**	NO	1.50	0.71	2.00	0.00							2.00		1.500	2	0.472
**TPUSHOUTT**	NO	1.30	0.48	1.50	0.55	1.20	0.45	1.22	0.44	1.75	0.96	1.20	0.45	2.991	5	0.701
**TATTSTDT**	NO	2.20	1.64	2.60	2.07	2.58	1.38	2.73	1.56	3.78	3.35	5.25	3.59	4.279	5	0.510
**TPREPT**	NO	12.47	9.16	10.77	7.40	8.70	4.62	8.33	4.64	11.85	7.93	11.87	9.32	2.373	5	0.795
**TATTGNDT**	NO	7.88	7.54	7.06	4.77	9.00	7.42	7.85	5.18	11.64	10.26	15.00	12.57	8.654	5	0.124
**TTRANST**	SÍ							8.00	2.83	8.00		4.50	3.39	1.122	2	0.385
**TNORPT**	NO	8.38	4.54	15.15	10.01	10.31	4.91	9.79	4.93	12.50	6.72	15.54	8.05	27.825	5	0.000
**TVIDPT**	SÍ	43.00				41.00						53.00	37.47	0.052	2	0.950
**TMEDPT**	SÍ			49.50	2.12							29.00		62.259	1	0.080
**TCHAPT**	SÍ	118.00										112.60	21.03	0.055	1	0.826
**TBKPT**	NO					48.08	6.01									
**TSTDS**	NO	2.34	0.67	1.87	1.05	1.27	0.50	2.05	0.83	1.43	0.70	1.63	0.88	54.649	5	0.000
**TGNDS**	NO	1.11	0.32	1.29	0.59	1.12	0.33	1.13	0.34	1.18	0.53	1.33	0.48	5.767	5	0.330
**TPS**	NO	1.41	0.54	2.03	1.06	1.90	0.84	1.34	0.55	1.50	0.69	1.77	0.97	16.733	5	0.005
**TNORPS**	NO	1.36	0.53	1.97	1.06	1.36	0.56	1.34	0.55	1.50	0.69	1.52	0.63	11.642	5	0.040
**TVIDPS**	NO	1.00				1.00						1.00	0.00	0.000	2	1.000
**TMEDPS**	NO			1.00	0.00							1.00		0.000	1	1.000
**TCHAPS**	NO	1.00										1.20	0.45	0.200	1	0.655
**TBKPS**	NO					1.00	0.00									
**STDST**	NO	25.30	9.55	37.84	20.16	44.77	17.26	31.19	15.72	42.44	18.42	31.75	19.24	28.421	5	0.000
**GNDST**	NO	15.21	13.11	11.36	4.85	12.03	7.21	12.00	6.54	17.58	13.48	17.04	12.27	4.238	5	0.516
**PST**	NO	7.60	9.33	8.73	5.00	5.31	3.18	7.44	3.26	8.57	3.30	18.66	20.82	57.422	5	0.000
**NORPST**	NO	6.02	1.99	7.85	2.79	7.80	2.80	7.44	3.26	8.57	3.30	10.65	4.84	32.009	5	0.000
**VIDPST**	SÍ	43.00				41.00						74.00	12.73	3.167	2	0.369
**MEDPST**	SÍ			49.50	2.12							29.00		62.259	1	0.080
**CHAPST**	SÍ	118.00										101.40	32.96	0.211	1	0.670
**BKPST**	NO					48.05	6.17									
**TNSEPA**	NO	6.70	2.98	5.16	1.93	4.79	2.25	5.10	2.11	4.41	1.60	4.71	2.04	18.014	5	0.003
**TNGRIP**	NO	6.45	2.83	5.33	1.99	4.98	2.15	4.95	1.95	4.77	2.18	4.23	2.12	17.401	5	0.004
**TNHOLD**	NO	1.26	0.54	1.50	0.67	1.32	0.48	1.52	0.73	1.57	0.95	2.33	1.31	16.734	5	0.005
**TNFALL**	NO	1.15	0.37	1.27	0.55	1.28	0.46	1.22	0.43	1.33	0.59	1.38	0.49	3.078	5	0.688
**TNFALL No Pt**	NO	1.07	0.27	1.27	0.47	1.11	0.33	1.09	0.30	1.25	0.62	1.37	0.50	6.253	5	0.282
**TNFALL 2 Pt**	NO	1.17	0.41	1.10	0.32	1.10	0.32	1.13	0.35	1.00	0.00	1.00	0.00	1.772	5	0.880
**TNFALL 4 Pt**	NO	1.00		1.00	0.00	1.00	0.00	1.00	0.00	1.00	0.00			0.000	4	1.000
**TNTHRO**	NO	1.00	0.00	1.00	0.00							1.00		0.000	2	1.000
**TNTHRO No Pt**	NO	1.00		1.00										0.000	1	1.000
**TNTHRO. 4 Pt**	NO	1.00		1.00								1.00		0.000	2	1.000
**TNPUSHOUT**	NO	1.00	0.00	1.00	0.00	1.00	0.00	1.00	0.00	1.25	0.50	1.00	0.00	9.250	5	0.099
**TNATTSTD**	NO	1.00	0.00	1.11	0.33	1.08	0.28	1.10	0.32	1.00	0.00	1.25	0.50	2.484	5	0.779
**TNPREP**	NO	2.43	1.45	1.83	0.72	1.44	0.88	1.64	1.21	1.69	0.95	1.27	0.46	8.617	5	0.125
**TNAGND**	NO	2.27	1.53	1.73	0.88	1.58	0.79	1.58	1.00	1.93	1.21	1.50	0.67	3.202	5	0.669
**TNAGND No Pt**	NO	1.14	0.38	1.30	0.67	1.14	0.38	1.00	0.00	1.43	0.79	1.27	0.47	2.029	5	0.845
**TNAGND 1Pt**	NO	1.00		1.00		1.00						1.00		0.000	3	1.000
**TNAGND 2Pt**	NO	2.50	1.18	1.38	0.52	1.29	0.49	1.56	1.13	1.70	1.06	1.20	0.41	10.655	5	0.059
**TNAGND Toc**	NO			1.00		1.00								0.000	1	1.000
**TNTRANS**	NO							1.00	0.00	1.00	0.00	1.00	0.00	0.000	2	1.000
**TNPA**	NO			1.00	0.00	1.00	0.00	1.00	0.00	1.00	0.00	1.00	0.00	0.000	4	1.000
**SEPAST**	NO	1.90	0.75	1.90	0.84	2.45	0.90	1.91	0.83	2.26	2.28	1.65	0.74	22.047	5	0.001
**GRIPST**	NO	6.88	4.12	7.30	3.86	9.70	11.22	9.20	9.04	7.87	4.55	6.72	4.62	8.757	5	0.119
**HOLDST**	NO	4.75	4.02	7.44	5.09	7.32	5.35	6.34	4.41	5.16	4.49	5.45	4.71	10.448	5	0.063
**FALLST**	NO	2.18	1.48	2.62	1.66	2.86	3.01	2.81	2.43	4.52	4.32	2.22	3.58	14.529	5	0.013
**THROWST**	NO	1.50	0.71	2.00	0.00							2.00		1.500	2	0.472
**PUSHOUTST**	NO	1.30	0.48	1.50	0.55	1.20	0.45	1.13	0.35	1.50	1.00	1.20	0.45	2.609	5	0.760
**ATTSTDST**	NO	2.50	1.73	2.28	1.52	2.38	1.15	2.60	1.07	3.14	2.12	4.00	1.83	4.036	5	0.544
**PREPST**	NO	5.18	3.04	6.44	4.00	7.48	2.70	6.48	4.51	8.93	8.20	9.60	8.02	4.295	5	0.508
**ATTGNDST**	NO	4.61	5.89	4.67	3.77	4.69	3.42	5.65	3.42	7.06	5.91	10.59	10.77	15.965	5	0.007
**TRANSST**	SÍ							8.00	2.83	8.00		5.20	3.27	0.723	2	0.530
**PAST**	NO			22.95	11.55	15.17	10.61	21.20	14.25	22.00	16.21	21.60	8.47	2.602	4	0.627

Note: Abbreviations in Table 3.

**Table 6:** Sequential and temporal parameters in the different minutes of the bout in the 125 kg category.

Categories	Nor	Moment in the bout												ANOVA or Kruskal-Wallis		
		1st minute		2nd minute		3rd minute		4th minute		5th minute		6th minute				
		Mean	SD	Mean	SD	Mean	SD	Mean	SD	Mean	SD	Mean	SD	F o H	gl	p
**TCT**	NO	66.26	4.97	74.48	35.63	77.07	21.24	74.34	20.21	80.54	28.14	77.00	33.41	10.727	5	0.057
**TFT**	NO	60.68	2.63	58.74	6.09	60.97	1.15	61.10	5.40	59.38	7.00	59.04	7.59	13.612	5	0.018
**TSTDT**	NO	55.26	10.23	53.55	13.01	56.31	7.46	54.21	11.50	52.81	9.30	50.36	11.66	8.528	5	0.129
**TGNDT**	NO	16.80	11.28	13.42	8.36	12.27	5.90	15.38	11.44	12.21	6.97	14.47	8.79	0.902	5	0.970
**TPT**	NO	6.41	3.94	16.83	37.72	19.46	21.81	16.70	19.77	23.91	27.90	24.94	35.27	29.481	5	0.000
**TPAT**	NO			27.88	10.70	23.60	10.81	19.80	13.37	29.82	16.70			2.027	3	0.567
**TSEPAT**	NO	11.29	6.04	9.17	5.60	10.76	6.05	10.17	5.88	10.50	6.63	9.08	5.51	3.108	5	0.683
**TGRIPT**	SÍ	39.87	12.91	38.27	10.69	39.45	10.69	39.48	13.21	37.00	12.23	31.28	11.35	1.947	5	0.089
**THOLDT**	NO	7.25	4.33	9.18	7.75	8.64	6.49	6.20	6.03	5.23	5.72	11.13	10.39	6.838	5	0.233
**TFALLT**	NO	2.80	2.15	2.20	0.92	2.38	1.51	2.00	1.25	3.42	3.55	2.33	1.40	1.104	5	0.954
**TTHROWT**	NO															
**TPUSHOUTT**	NO	1.00	0.00	1.40	0.89	1.50	0.84	2.00	0.00	1.67	1.15	1.00	0.00	6.281	5	0.280
**TATTSTDT**	NO	2.33	0.58	4.80	4.15	6.75	3.59	2.00	0.00	3.83	2.23	3.56	3.40	4.195	5	0.522
**TPREPT**	SÍ	11.22	7.40	8.11	6.70	8.17	6.31	7.50	6.55	5.82	3.25	9.67	4.42	0.986	5	0.437
**TATTGNDT**	NO	7.11	5.06	6.70	5.01	6.75	4.56	10.67	9.83	8.25	5.88	9.45	6.65	1.456	5	0.918
**TTRANST**	SÍ			7.33	2.89	11.00	2.00	6.50	4.95	6.00		7.00	3.74	0.889	4	0.512
**TNORPT**	NO	6.41	3.94	9.90	5.29	11.09	6.76	11.29	4.63	16.00	8.67	12.76	6.35	27.035	5	0.000
**TVIDPT**	NO					50.00										
**TMEDPT**	SÍ					45.00		50.00		51.00					2	
**TCHAPT**	SÍ			202.00		64.00	19.80	97.00		97.00	9.90	116.50	21.92	10.203	4	0.043
**TBKPT**	NO					50.93	8.86									
**TSTDS**	NO	2.06	0.63	1.45	0.62	1.38	0.73	2.17	0.80	1.62	0.80	1.44	0.77	35.411	5	0.000
**TGNDS**	NO	1.10	0.32	1.00	0.00	1.09	0.30	1.29	0.47	1.07	0.27	1.47	0.64	10.598	5	0.060
**TPS**	NO	1.22	0.51	1.45	0.63	2.24	0.99	1.39	0.58	1.61	0.89	1.44	0.51	28.256	5	0.000
**TNORPS**	NO	1.22	0.51	1.41	0.63	1.45	0.67	1.43	0.60	1.62	0.86	1.41	0.51	4.204	5	0.520
**TVIDPS**	NO					1.00										
**TMEDPS**	NO					1.00		1.00		1.00				0.000	2	1.000
**TCHAPS**	NO			1.00		1.00	0.00	1.00		1.00	0.00	1.00	0.00	0.000	4	1.000
**TBKPS**	NO					1.00	0.00									
**STDST**	NO	29.50	12.26	41.86	17.25	48.28	16.62	29.55	15.42	39.99	17.92	41.61	16.98	25.008	5	0.000
**GNDST**	NO	15.65	11.17	14.70	8.63	11.32	5.15	10.71	6.86	11.57	6.81	9.50	3.84	3.935	5	0.559
**PST**	NO	5.05	1.59	10.42	18.46	7.37	8.44	13.98	20.32	15.97	19.65	17.92	25.43	37.597	5	0.000
**NORPST**	NO	5.05	1.59	7.13	2.61	7.61	3.14	8.23	3.37	9.48	4.64	9.35	5.14	27.210	5	0.000
**VIDPST**	NO					50.00										
**MEDPST**	NO					45.00				51.00				1.000	1	0.317
**CHAPST**	SÍ			202.00		64.00	19.80	97.00		97.00	9.90	116.50	21.92	10.203	4	0.043
**BKPST**	NO					50.93	8.86									
**TNSEPA**	NO	5.71	2.42	4.53	2.16	4.83	1.83	5.45	2.13	4.38	1.65	4.72	2.21	7.191	5	0.207
**TNGRIP**	NO	5.68	2.20	4.93	1.95	4.55	1.70	5.45	1.94	4.38	1.55	4.84	2.06	7.765	5	0.170
**TNHOLD**	NO	1.67	0.78	1.68	0.84	1.40	0.70	1.27	0.46	1.27	0.47	1.81	1.11	5.382	5	0.371
**TNFALL**	NO	1.09	0.30	1.00	0.00	1.00	0.00	1.27	0.46	1.15	0.38	1.33	0.62	7.067	5	0.216
**TNFALL No Pt**	NO	1.00	0.00	1.00	0.00	1.00	0.00	1.14	0.38	1.00	0.00	1.40	0.70	7.036	5	0.218
**TNFALL 2 Pt**	NO	1.00	0.00	1.00	0.00	1.00	0.00	1.00	0.00	1.00	0.00	1.00	0.00	0.000	5	1.000
**TNFALL 4 Pt**	NO	1.00		1.00	0.00			1.00	0.00	1.00	0.00			0.000	3	1.000
**TNTHRO**	NO															
**TNTHRO No Pt**	NO															
**TNTHRO. 4 Pt**	NO															
**TNPUSHOUT**	NO	1.00	0.00	1.00	0.00	1.00	0.00	1.00	0.00	2.00	1.41	1.33	0.58	7.702	5	0.173
**TNATTSTD**	NO	1.00	0.00	1.00	0.00	1.00	0.00	1.00	0.00	1.17	0.41	1.25	0.71	1.974	5	0.853
**TNPREP**	NO	2.11	1.27	1.75	1.04	1.33	0.52	1.44	0.73	1.40	0.97	1.33	0.50	5.265	5	0.384
**TNAGND**	NO	1.89	1.05	1.67	1.12	1.43	0.79	1.36	0.67	1.42	0.90	1.73	1.01	2.969	5	0.705
**TNAGND No Pt**	NO	1.25	0.50	1.00	0.00	1.00	0.00	1.11	0.33	1.75	1.50	1.67	0.82	7.023	5	0.219
**TNAGND 1Pt**	NO	1.50	0.71	1.00				1.00		1.00	0.00			2.000	3	0.572
**TNAGND 2Pt**	NO	1.50	0.55	2.67	1.15	1.67	1.15	1.33	0.58	1.00	0.00	1.33	0.52	10.741	5	0.057
**TNAGND Toc**	NO							1.00	0.00			1.00		0.000	1	1.000
**TNTRANS**	NO			1.33	0.58	1.00	0.00	1.00	0.00	1.00		1.25	0.50	1.955	4	0.744
**TNPA**	NO			1.00	0.00	1.00	0.00	1.00	0.00	1.00	0.00			0.000	3	1.000
**SEPAST**	NO	2.05	0.90	2.02	0.80	2.31	1.32	1.83	0.85	2.35	0.98	1.96	0.94	5.930	5	0.313
**GRIPST**	NO	7.94	4.24	9.27	5.63	9.45	3.40	8.21	4.67	10.00	6.32	7.68	4.52	7.421	5	0.191
**HOLDST**	NO	4.56	2.78	5.39	3.79	6.78	4.22	4.90	3.71	2.64	1.57	6.26	5.23	10.703	5	0.058
**FALLST**	NO	2.40	1.26	2.20	0.92	2.38	1.51	1.77	1.35	2.71	2.18	1.94	1.39	4.155	5	0.527
**THROWST**	NO															
**PUSHOUTST**	NO	1.00	0.00	1.40	0.89	1.20	0.45	2.00	0.00	1.00	0.00	0.83	0.29	9.431	5	0.093
**ATTSTDST**	NO	2.33	0.58	4.80	4.15	6.00	4.00	2.00	0.00	3.67	2.42	3.50	3.70	2.592	5	0.763
**PREPST**	NO	5.87	4.12	4.63	4.22	6.42	5.95	5.63	6.21	5.73	3.65	7.25	3.78	3.289	5	0.656
**ATTGNDST**	NO	3.94	2.40	5.14	5.43	6.33	4.77	8.17	7.30	6.46	5.28	5.64	3.32	2.946	5	0.708
**TRANSST**	SÍ			5.83	2.75	11.00	2.00	6.50	4.95	6.00		5.50	1.73	2.208	4	0.158
**PAST**	NO			27.88	10.70	23.60	10.81	19.80	13.37	30.50	17.44			2.155	3	0.541

Note: Abbreviations in Table 3.

## Discussion

### General discussion about the bout

The significant differences found in the temporal and sequential parameters of the freestyle wrestling bout were very numerous among the three weight categories. In contrast, other authors [13] did not find any differences when comparing weight categories. This is because these authors grouped the weight categories together when making comparisons. In our study, as in other studies in judo [5, 22], we did not group weight categories. We believe that it is not always a good measure to group weight categories, as it may lead to a loss of information.

With respect to the total time of the bout, we observed quite a bit of similarity among the three categories. This trend was found by other authors [13]. Moreover, the 433 seconds [13] and the 440 seconds [3] detected in previous studies appear in the results of our research (422–459 seconds).

In the total fighting time (excluding interruptions), we did not find any significant differences between the three categories. Other authors also did not find any differences when comparing weight categories [13]. However, there are authors who did find differences [12]. When we compared our 125 kg results with other studies, we detected a difference of about 30 seconds [11–13].

Similar to other authors [13], there are no differences in total pause time between weight categories. Moreover, the mean pause values (128 seconds) of these authors are between those found in our study (114–132 seconds).

With respect to the 30 seconds of regulatory break, we found that this time was considerably longer in the three categories (50 seconds in 65 kg, 48 seconds in 86 kg and 51 seconds in 125 kg). Other authors also noted this tendency [13], although to a lesser extent (41 seconds in light-weight, 44 seconds in middle-weight and 43 seconds in heavy-weight).

Regarding the standing wrestling, we can highlight that the higher the weight category, the more time the competitors spend wrestling in a standing situation because they perform longer standing wrestling sequences. It should be noted that this tendency is not observed in other authors [13], where they found 38 seconds in light-weight and middle-weight and 39 seconds in heavy-weight, while we obtained 27 seconds in 65 kg, 32 seconds in 86 kg and 36 seconds in 125 kg. These differences are probably due to the fact that the aforementioned authors did not differentiate between standing and ground wrestling.

Competitors weighing 65 kg are the ones who spend the most time separated when wrestling while standing, because when they perform a separate action, it clearly lasts longer. On the contrary, the higher the weight category, the more time competitors spend gripping. Thus 125 kg wrestlers are the ones who spend the most time in a grip because when they grip, they are the ones who spend the most time searching for a technical action.

Competitors weighing 125 kg spent the least amount of time performing falls because these competitors clearly performed the fewest number of falls. This is a circumstance that resembles the results achieved in recent studies [13].

The lower the weight category, the more time competitors spend wrestling on the ground. Competitors weighing 65 kg clearly spend the most time because they perform a greater number of wrestling sequences on the ground. This is consistent with previous studies, where light-weight competitors perform a greater number of wrestling sequences [13].

In addition, the total duration of attacks performed by competitors on the ground is also longer the lower the weight category, which was a trend that was noted by the other authors [13]. Competitors weighing 65 kg spend the most time because they perform almost twice as many attacks as competitors in the remaining categories.

### Discussion according to the bout minute

The analysis of the data as a function of the minutes of the bout has been carried out in disciplines such as judo [22] but has not been carried out in freestyle wrestling, which makes it difficult to discuss the results.

In the three weight categories, multiple significant differences in the temporal and sequential parameters were obtained when comparing the different minutes of the bout. The 65 and 85 kg categories stand out with double the differences compared to the 125 kg category. It is therefore necessary to discuss the results in each of the weight categories.

In 65 kg we can highlight that as the bout progresses, the total duration of the minutes is longer (it must be considered that the pauses are included), with a small stabilization between the third and fourth minute due to the regulatory break. This is because the same behavior follows the total pause time.

At 65 kg, the ratio of standing wrestling time to ground wrestling time is 74% vs. 26%, which is similar data from other authors [13]. Clearly, wrestlers spend more time wrestling while standing. In the first minute, most of the time fighting is spent while standing, while in the last minute they spend the least. This could be because the first minute is where more standing sequences occur.

In 65 kg we observed coherence in the pauses of the bout, occurring at the beginning of the bout where there are fewer pauses and where the pauses are shorter, increasing both aspects as the bout progresses, suffering a reasonable decrease in the fourth minute of the bout (just after the regulatory break), to later increase gradually until experiencing a drastic increase in the last minute, as a result of accumulated fatigue. This behavior in the last minute of the combat has also been found in judo [22].

In 86 kg the total duration of the minutes is increased in the first two minutes of the bout. In the third to fifth minutes the increase is slight (probably due to the regulatory break) and in the last minute the increase is drastic (about 50%). We can probably associate this increase in the duration of the minutes to the duration of the pauses that occur during each minute of the bout, the duration of the pause sequences in the last minute being twice as long as in the rest of the minutes, probably as a strategy to recover their breath due to accumulated fatigue. This strategy is also detected in judo [22].

In 86 kg, the ratio of time spent wrestling standing to time spent wrestling on the ground is 75% vs. 25%, very similar to 65 kg and similar to that found by other authors [13]. As in 65 kg, the first minute is the one in which they spend more time wrestling standing, and the last minute is the one in which they spend less time wrestling while standing. That is probably because the first minute is where the most standing sequences occur.

At 86 kg we will highlight that the time of a ground attack is clearly higher in the last minute of the bout. This explains why the total time of ground attacks is higher in the last minute, and why the last minute the proportion of ground fighting rises to 34%.

At 125 kg, the proportion of time spent wrestling standing to time spent wrestling on the ground is 79% versus 21%. It is in this weight category that standing wrestling is most commonly used, which is a circumstance that has also been found in recent studies [13]. It should be noted that in the first minute of the bout and in the minute after the regulatory break is where there are more sequences of standing fights. It is precisely in these minutes that the duration of a standing sequence is clearly lower than in the rest of the minutes. Both circumstances would explain why the total standing time is so similar in the different minutes of the bout.

In 125 kg, at the beginning of the bout, there are fewer pauses, the pauses are shorter, and the total pause time is less, increasing these three aspects as the bout progresses, while having a decrease in the fourth minute of the bout (just after the regulatory break), and then gradually increasing until the end of the bout, which could be explained by the accumulation of fatigue. This accumulation is also found in judo [22].

### Limitations

There are not any studies where the temporal parameters of freestyle wrestling are analyzed based on the different minutes of the bout. This aspect, together with the grouping of the weight categories [13] or the presentation of the data in a global form [11] has made it difficult to discuss the results. The differences found between the three weight categories indicates that it is necessary to replicate this study in the other weight categories.

### Practical applications

The results of this study show clear differences between the three weight categories. This implies that the results cannot be generalized. They must be individualized by weight. With the results of the study, we have elaborated a temporal bout structure model for each of the categories: 65 kg (Table 7), 86 kg (Table 8) and 125 kg (Table 9).

**Table 7:** Temporal structure model of a freestyle wrestling bout in the 65 kg male category.

**Sequence**	**Action**	**t (s)**	**Sequence**	**Action**	**t (s)**	**Sequence**	**Action**	**t (s)**
**1st minute**			**2nd minute**			**3rd minute**		
STD 1		25.6	Pause 2 normal		6.73	STD 5		41.90
	Separation	3.47	STD 3		30.70		Grip	6.60
	Grip	5.02		Separation	3.15		Separation	4.61
	Separation	3.47		Grip	6.05		Grip	6.60
	Grip	5.02		Separation	3.15		Separation	4.61
	Separation	3.47		Hold	5.68		Hold	6.76
	Grip	5.02		Grip	6.05		Separation	4.61
	STD Attack	2.56		Hold	5.68		Grip	6.60
	Push-out	1.25		Fall no PT	1.76	Pause 4 normal		8.71
Pause 1 normal		5.6	GND 2		12.47	Passivity		20.72
STD 2		25.6		Attack 2 points	5.60	STD 6		41.90
	Separation	3.47		Preparation	6.42		Separation	4.61
	Grip	5.02	Pause 3 normal		6.73		Grip	6.60
	Hold	5.38	STD 4				Separation	4.61
	Grip	5.02		Separation	3.15		Grip	6.60
	Separation	3.47		Grip	6.05		Fall no PT	2.19
	Grip	5.02		Separation	3.15	GND 4		11.64
	Separation	3.47		Grip	6.05		Attack no point	5.49
	Hold	5.38		Separation	3.15		Preparation	7.07
	Fall no PT	1.24		Grip	6.05	Pause 5 Break		49.62
GND 1		10.73		Fall no PT	1.76			
	Attack 2 points	5.98	GND 3		12.47			
	Preparation	6.39		Attack no point	5.60			
				Transition	10.00			
**Sequence**	**Action**	**t (s)**	**Sequence**	**Action**	**t (s)**	**Sequence**	**Action**	**t (s)**
**4th minute**			**5th minute**			**6th minute**		
STD 7		24.22	Pause 8 normal		8.52	Pause 10 normal		27.16
	Separation	2.70	STD 10		32.78	STD 12		29.10
	Grip	4.88		Separation	3.19		Separation	3.29
	Separation	2.70		Grip	6.00		Grip	5.14
	Hold	6.23		Separation	3.19		Separation	3.29
	Fall no PT	1.96		STD Attack	3.21		Grip	5.14
GND 5		15.39		Grip	6.00		Hold	5.17
	Attack no PT	7.95		Hold	5.49		Fall no PT	1.43
	Preparation	9.16		Fall no PT	1.81	GND 8		11.18
	Attack 2 points	7.95	GND 6		13.01		Attack no point	5.84
Pause 6 normal		7.47		Preparation	7.42		Preparation	7.64
STD 8		24.22		Attack no point	5.59		Attack 1 point	5.84
	Separation	2.70	Pause 9 normal		8.52		Transition	2.40
	Grip	4.88	STD 11		32.78	Pause 11 reclamación		121.87
	Separation	2.70		Separation	3.19	STD 13		29.10
	Grip	4.88		Grip	6.00		Separation	3.29
	Hold	6.23		Hold	5.49		Grip	5.14
	Grip	4.88		Separation	3.19		Separation	3.29
	Throw 4 PT	1.00		Grip	6.00		Grip	5.14
Pause 7 normal		7.47		Fall no PT	1.81		Hold	5.17
STD 9		24.22	GND 7		13.01			
	Separation	2.70		Attack 2 points	5.59			
	Grip	4.88		Preparation	7.42			
	Separation	2.70		Transition	4.11			
	Grip	4.88						
	STD Attack	5.22						
	Push-out	1.00						

Abbreviations: t = time; STD = Standing; GND = Ground; PT = point.

**Table 8:** Temporal structure model of freestyle wrestling bout in the 86 kg male category.

**Sequence**	**Action**	**t (s)**	**Sequence**	**Action**	**t (s)**	**Sequence**	**Action**	**t (s)**
**1st minute**			**2nd minute**			**3rd minute**		
STD 1		25.30	STD 3		37.84	STD 5		44.77
	Separation	1.90		Separation	1.90		Separation	2.45
	Grip	6.88		Grip	7.30		Grip	9.70
	Separation	1.90		STD Attack	2.28		Separation	2.45
	Grip	6.88		Separation	1.90		Grip	9.70
	Separation	1.90		Grip	7.30		Hold	7.32
	Grip	6.88		Fall no PT	2.62		STD Attack	2.38
	Separation	1.90	GND 2		11.36		Separation	2.45
Pause 1 normal		6.02		Attack no point	4.67		Grip	9.70
STD 2		25.30		Preparation	6.44		Separation	2.45
	Separation	1.90		Attack 2 points	4.67		Grip	9.70
	Grip	6.88		Preparation	6.44		Separation	2.45
	Separation	1.90	Pause 3 normal		7.85		Grip	9.70
	Grip	6.88	STD 4		37.84		Fall no PT	2.86
	Separation	1.90		Separation	1.90	GND 3		12.03
	Grip	6.88		Grip	7.30		Attack 2 points	4.69
	Hold	4.75		Hold	7.44		Preparation	7.48
	Fall 2 PT	2.18		Separation	1.90	Pause 5 Break		48.05
GND 1		15.21		Grip	7.30			
	Preparation	5.18		Separation	1.90			
	Attack 2 points	4.61		Grip	7.30			
	Preparation	5.18		Hold	7.44			
	Attack no point	4.61		Push-out	1.50			
Pause 2 normal		6.02	Pause 4 normal		7.85			
**Sequence**	**Action**	**t (s)**	**Sequence**	**Action**	**t (s)**	**Sequence**	**Action**	**t (s)**
**4th minute**			**5th minute**			**6th minute**		
STD 6		31.19	Pause 7 normal		8.57	STD 9		31.75
	Separation	1.91	Passivity		20.50		Separation	1.65
	Grip	9.20	STD 8		42.44		Grip	6.72
	Separation	1.91		Separation	2.26		Hold	5.45
	Grip	9.20		Grip	7.87		Separation	1.65
	Separation	1.91		Hold	5.16		Grip	6.72
	Hold	6.34		Grip	7.87		Push-out	1.20
	Grip			Separation	2.26	Pause 9 Challenge		101.40
	Fall 2 PT	2.81		Grip	7.87	STD 10		31.75
GND 4		12.00		Separation	2.26		Separation	1.65
	Preparation	6.48		Grip	7.87		Grip	6.72
	Attack no point	5.65		Separation	2.26		Separation	1.65
	Preparation	6.48		Grip	7.87		Grip	6.72
	Attack 2 points	5.65		Fall no PT	4.52		Throw 4 PT	2.00
Pause 6 normal		7.44	GND 5		17.58	GND 6		17.04
STD 7		31.19		Attack no point	7.06		Preparation	9.60
	Separation	1.91		Preparation	8.93		Attack no point	10.59
	Hold	6.34		Attack 2 points	7.06			
	Grip	9.20		Preparation	8.93			
	Separation	1.91		Transition	8.00			
	Grip	9.20	Pause 8 normal		8.57			
	Push-out	1.13						

Abbreviations: t = time; STD = Standing; GND = Ground; PT = point.

**Table 9:** Temporal structure model of a freestyle wrestling bout in the 125 kg male category.

**Sequence**	**Action**	**t (s)**	**Sequence**	**Action**	**t (s)**	**Sequence**	**Action**	**t (s)**
**1st minute**			**2nd minute**			**3rd minute**		
STD 1		29.50	STD 3		41.86	STD 5		48.28
	Separation	2.05		Separation	2.02		Separation	2.31
	Grip	7.94		Grip	9.27		Grip	9.45
	Separation	2.05		Hold	5.39		Separation	2.31
	Grip	7.94		Grip	9.27		Hold	6.78
	Separation	2.05		Separation	2.02		Grip	9.45
	Grip	7.94		Grip	9.27		Separation	2.31
	Separation	2.05	Pause 2 normal		10.42		Grip	9.45
	Grip	7.94	Passivity		27.88		Separation	2.31
	Push-out	1.00	STD 4		14.70		Grip	9.45
Pause 1 normal		5.05		Separation	2.02		Hold	6.78
STD 2		29.50		STD Attack	4.80		Separation	2.31
	Separation	2.05		Grip	9.27		Grip	9.45
	Grip	7.94		Separation	2.02		Fall no PT	2.38
	Separation	2.05		Grip	9.27	GND 3		11.32
	Grip	7.94		Fall no PT	2.20		Attack no point	6.33
	Hold	4.56	GND 2		14.70		Preparation	6.42
	Fall no PT	2.40		Preparation	4.63		Attack 2 points	6.33
GND 1		15.65		Attack no point	5.14	Pause 4 Break		50.93
	Preparation	5.87		Preparation	4.63			
	Attack 2 points	3.94		Attack 2 points	5.14			
	Preparation	5.87		Transition	5.83			
	Attack no point	3.94	Pause 3 normal		10.42			
**Sequence**	**Action**	**t (s)**	**Sequence**	**Action**	**t (s)**	**Sequence**	**Action**	**t (s)**
**4th minute**			**5th minute**			**6th minute**		
STD 6		29.55	Pause 6 medical		51.00	Pause 8 normal		9.35
	Separation	1.83	STD 8		39.99	STD 10		41.61
	Grip	8.21		Separation	2.35		Separation	1.96
	Separation	1.83		Grip	10.00		Grip	7.68
	Grip	8.21		Separation	2.35		Separation	1.96
	Push-out	2.00		Grip	10.00		Hold	6.26
Pause 5 normal		8.23		Hold	2.64		Grip	7.68
STD 7		29.55		Push-out	1.00		Separation	1.96
	Separation	1.83	Pause 7 normal		9.48		STD Attack	3.50
	Grip	8.21	STD 9		39.99		Grip	7.68
	Hold	4.90		Separation	2.35		Fall no PT	1.94
	Separation	1.83		Grip	10.00	GND 6		9.50
	Grip	8.21		Separation	2.35		Attack no point	5.64
	Separation	1.83		Grip	10.00		Preparation	7.25
	Grip	8.21		Fall 2 PT	2.71		Attack 2 points	5.64
	Fall no PT	1.77	GND 5		11.57	Pause 9 normal		9.35
GND 4		10.71		Attack no point	6.46	STD 11		41.61
	Preparation	5.63		Preparation	5.73		Separation	1.96
	Attack no point	8.17		Transition	6.00		Grip	7.68
							Hold	6.26

Abbreviations: t = time; STD = Standing; GND = Ground; PT = point.

## Conclusions

We propose a temporal bout structure model for freestyle wrestling per each weight. Based on the data, sports performance professionals will be able to develop more accurate and appropriate training programs.

In the three weight categories, the wrestlers spend more time fighting while standing (76%) than ground wrestling (24%). The 125 kg wrestlers fight more time while standing, are gripping for longer and they are the ones who perform the fewest falls. Competitors weighing 65 kg spend the most time separated. The 65 kg wrestlers spend the most time wrestling on the ground due to twice as many attacks.

The 125 kg category has some more stable temporal and sequential parameters throughout the different minutes of the bout. The 65 and 86 categories are characterized by the instability of those parameters throughout the different bout minutes.

In the three weight categories, the first bout minute has fewer pause time, and the last minute has more pause time due to the accumulated fatigue throughout the bout.

In the three weight categories, the regulatory break modulates the duration of the pauses of the different minutes of the bout and the actions performed by the wrestlers.
